# A recombinant porcine reproductive and respiratory syndrome virus type 2 field strain derived from two PRRSV-2-modified live virus vaccines

**DOI:** 10.3389/fvets.2023.1149293

**Published:** 2023-03-28

**Authors:** Giovani Trevisan, Drew Magstadt, Amy Woods, Joel Sparks, Michael Zeller, Ganwu Li, Karen M. Krueger, Anugrah Saxena, Jianqiang Zhang, Phillip C. Gauger

**Affiliations:** ^1^Veterinary Diagnostic and Production Animal Medicine, Iowa State University, Ames, IA, United States; ^2^AMVC, Frankfort, IN, United States; ^3^Programme in Emerging Infectious Diseases, Duke-NUS Medical School, Singapore, Singapore

**Keywords:** PRRSV, NGS, MLV vaccines, recombination, evolution

## Abstract

A porcine reproductive and respiratory syndrome virus (PRRSV) type 2 (PRRSV-2) isolate was obtained from lung samples collected from a 4.5-month-old pig at a wean-to-finish site in Indiana, USA, although no gross or microscopic lesions suggestive of PRRSV infection were observed in the lung tissue. Phylogenetic and molecular evolutionary analyses based on the obtained virus sequences indicated that PRRSV USA/IN105404/2021 was a natural recombinant isolate from Ingelvac PRRS^®^ MLV and Prevacent^®^ PRRS, which are PRRSV-2-modified live virus vaccines commercially available in the United States. This study is the first to report the detection of a PRRSV-2 recombinant strain consisting entirely of two modified live virus vaccine strains under field conditions. Based on clinical data and the absence of lung lesions, this PRRSV-2 recombinant strain was not virulent in swine, although its pathogenicity needs to be confirmed by clinical trials.

## 1. Introduction

Porcine reproductive and respiratory syndrome virus (PRRSV) is the etiologic agent of PRRS, a systemic swine disease (1). The PRRSV was first isolated in the early 1990s at a similar time in North America (USA) and Europe (the Netherlands) (2, 3). Since the emergence of PRRSV, the virus has continued to spread globally (1). The PRRSV is an enveloped, single-stranded, positive-sense RNA virus of the order Nidovirales and family Arteriviridae (4) and is classified into two distinct species: *Betaarterivirus suid 1* which is colloquially known as PRRSV-1 or the European type, and *Betaarterivirus suid 2* which is colloquially known as PRRSV-2 or the North American type (3–9). The entire PRRSV genome comprises ~ 15.1 kilobases consisting of at least 10 open reading frames (ORFs). Between the two PRRSV species, the ORF5 gene shares approximately 60% nucleotide identity (1, 4, 10).

Porcine reproductive and respiratory syndrome (PRRS) is one of the major swine diseases, causing significant economic losses associated with infection and clinical disease. Annual losses attributed to PRRSV are $664 million in the USA (11), $150 million in Canada (12), and between €75.72 and €650.09 per sow in Europe (13). The major clinical problem associated with PRRSV is reproductive failure in sows and respiratory distress, primarily observed not only in growing pigs but also in adult swine (1). Immunization with PRRS-modified live virus (MLV) vaccines has been used as a strategy to reduce the impact associated with PRRSV systemic disease and seeks to reduce clinical signs, viral shedding, and reproductive and respiratory losses associated with infection (14–16). PRRS MLV vaccines are the predominant immunological solution(s) used in breeding herds in the USA, with an increased number of production systems using this method of exposure (17–20). The use of PRRS MLV vaccines in breeding herds does not cause significant changes in production parameters following the first PRRS MLV exposure (21, 22).

Co-circulation of multiple PRRSV strains in a breeding herd is a reality (23). The emergence of new PRRSV strains can be due to low fidelity during RNA virus replication, recombination events, or random mutations (1, 24). A recombination event can occur between wild-type vs. wild-type PRRSV strains, wild-type vs. PRRSV MLV vaccine strains, or PRRSV MLV vaccine vs. PRRSV MLV vaccine strains. Co-infection by two or more strains in the same host and cell is a prerequisite for the occurrence of a recombination event (25, 26). Novel pathogenic PRRSV-2 strains derived from recombining wild-type and PRRSV MLV vaccine strains have been reported in China (27–29). PRRSV-2 recombinant strains derived from wild-type vs. PRRSV MLV vaccine strains have been reported in the USA (23, 30, 31) and Canada (32). PRRSV-2 recombinant strains derived from two wild-type strains have also been reported in the United States (23). In Denmark and France, it has been reported that pathogenic PRRSV-1 strains originated from the recombination between two PRRSV-1 MLV vaccine strains (33–35). Nevertheless, recombination between two PRRSV-2 MLV vaccine strains has not been reported in the USA. This study reports the detection of a recombinant PRRSV-2 isolate derived from two commercially available PRRSV-2 MLV vaccines in the USA under field conditions.

## 2. Methods

### 2.1. Overview of the clinical case and PRRS MLV exposure

At a wean-to-finish site located in Indiana, USA, where 4,882 4.5-month-old pigs were initially housed, a clinical history of coughing and increased mortality was observed. Overall wean-to-finish closeout mortality reached 21.06% (1,028 of 4,882). An assorted set of tissues (lung, spleen, and lymph nodes) were collected from three pigs, and one lung sample was collected individually from one pig (hereon referred to as “lung”). In addition, oral fluid samples (*n* = 2) were also collected. Samples were received at the Iowa State University Veterinary Diagnostic Laboratory (ISU-VDL) for routine disease investigation and diagnosis at the beginning of December 2021. Wean-to-finish piglets originating from a single sow farm flow had a history of endemic respiratory pathogens, including *Mycoplasma hyopneumoniae* (MHP) and influenza A virus (IAV), and were exposed to Prevacent^®^ PRRS MLV vaccine, following the production system vaccination protocol. All breeding females were intramuscularly vaccinated with Ingelvac PRRS^®^ MLV (GenBank AF066183) on 6 January 2021 in response to a PRRSV outbreak that had occurred in November 2020. Weaning-age pigs were placed in the wean-to-finish site and were intramuscularly vaccinated with Prevacent^®^ PRRS (GenBank KU131568) between 16 August 2021 and 24 August 2021, i.e., at placement time.

### 2.2. Diagnostic testing

The assorted set of tissues, the individual lung sample, and oral fluid samples was submitted for ancillary testing, including routine bacterial culture evaluation, real-time polymerase chain reaction (rtPCR) or reverse transcription-real-time PCR (RT-rtPCR) testing (collectively referred to as PCR), and histopathology (assorted tissues and individual lung sample). Tissues and oral fluid samples were tested for PRRSV-1, PRRSV-2, IAV, MHP, African swine fever, and classical swine fever by PCR assays routinely used at the ISU-VDL. Specifically, the commercial PRRSV PCR, VetMAX^TM^ PRRSV NA&EU Reagent (Thermo Fisher Scientific), targeting the conserved ORF6 and ORF7 genomic regions, was used as a screening PCR to detect and distinguish PRRSV-1 and PRRSV-2 in samples.

Upon a PRRSV-2 PCR positive result, the individual lung sample was tested using Ingelvac PRRS^®^ MLV vaccine-like PCR (targeting the nsp2 region) and Prevacent^®^ PRRS vaccine-like PCR (targeting the nsp2 region), as previously described (36). A PRRSV ORF5 CLAMP sequencing assay, targeting the conserved ORF5 genomic region, was applied to investigate the presence of a wild type PRRSV strain. CLAMP blocks the targeted vaccine ORF5 amplification and enhances the amplification of wild-type sequences that may be present in lower concentrations, with positive results indicating the presence of a wild-type PRRSV strain in the sample and negative results indicating the presence of a vaccine-like PRRSV strain (36, 37). PRRSV open reading frame 5 (ORF5) sequencing was performed using the Sanger technique (38, 39). PRRSV virus isolation (VI) was attempted from the lung sample following the previously described procedures (40). In brief, a 10% lung homogenate was prepared, filtered through a 0.45 μm pore-size filter, and inoculated (200 μl) into each cell of 24-well plates containing either MARC-145 or ZMAC cells. The cell plates were incubated for 1 h at 37°C with 5% CO_2_, after which the inoculum was removed and 1 ml of cell culture medium was added to each well. MARC-145 cells were examined daily for up to 5 days and ZMAC cells for 3 days for the development of cytopathic effect (CPE), after which the cell culture supernatants were harvested and the plates were fixed with 80% cold acetone for 10 min at room temperature, followed by immunofluorescence staining using PRRSV nucleocapsid protein-specific monoclonal antibodies (Rural Technologies Inc., South Dakota, USA) to detect the presence of PRRSV (40). If the first passage (P0) was negative, supernatants were inoculated into the respective cell lines for the second passage (P1). VI was considered negative if no growth was observed after two passages (40). Indigenous lung samples and the virus isolate obtained in ZMAC cells (P0) were both submitted for PRRSV whole genome sequencing *via* next-generation sequencing (NGS) technology, as previously described (39). All tests were performed on a fee-for-service basis at ISU-VDL.

### 2.3. PRRSV genetic comparisons and recombination analysis

The complete PRRSV genome sequence recovered from the virus isolate USA/IN105404/2021 was compared with a selected number of US wild-type and commercially available PRRSV MLV vaccine strains. The commercial PRRSV MLV vaccines currently available in the USA included in the comparison are Fostera^®^ PRRS (GenBank AF494042), Ingelvac PRRS^®^ MLV (GenBank AF066183), Ingelvac PRRS^®^ ATP (GenBank DQ988080), Prevacent^®^ PRRS (GenBank KU131568), Prime Pac^®^ PRRS (GenBank DQ779791), and PRRSGard^®^ (ISU-VDL in house vaccine bottle recovered sequence).

For sequence comparison and phylogenetic analysis, the NGS sequence recovered in this study, PRRS MLV vaccine sequences, and wild-type strain whole genome sequences were uploaded in Geneious Prime 2022.1.1 (https://www.geneious.com) and aligned using MAFFT v7.450 default settings (41, 42). The aligned sequences were used to construct a phylogenetic tree. Using default settings, a neighbor-joining consensus tree was built using the Tamura–Nei genetic distance with a bootstrap resampling method run with 100 replications in Geneious Tree Builder. Genes (ORF1-7) and non-structural proteins (nsp1-12) were annotated based on the ATCC VR-2332 strain (GenBank U87392.3), extracted and aligned using the MAFFT v7.450, and pairwise distances computed between USA/IN105404/2021 and all other compared sequences.

The investigation of the recombination event followed a previously described methodology (23). Aligned sequences were entered and used to detect recombination events and breakpoints using the recombination detection program RDP4 software version v.4.101 (43). A recombination event was considered if it was supported by at least six of the seven available RDP4 default parameters, namely, RDP, GENECONV, BootScan, MaxChi, Chimera, SiScan, and 3Seq. The complete PRRSV sequence with detected recombination events was further plotted using SimPlot version 3.5.1 (44) to confirm and visualize recombination breakpoints using a 200-bp window that slides with the genome alignment (20-bp step size).

## 3. Results

Ancillary diagnostic testing in oral fluid samples was PCR negative for IAV and PRRSV-1 and PCR positive for PRRSV-2 (Ct 36.0 and 35.7) and MHP (Ct 32.7 and 33.0). The spleen was PCR negative for classical swine fever virus and African swine fever virus. There was moderate growth of *Streptococcus suis* in the lung. The lung sample was negative by IAV PCR, PRRSV-1 screening PCR, and PRRSV Ingelvac MLV vaccine-like PCR but positive by MHP PCR (Ct 21.6). In addition, the lung was PRRSV-2 screening PCR positive (Ct 15.2) and PRRSV Prevacent vaccine-like PCR positive (Ct 18.0). Sanger sequencing was performed two times on the lung sample and recovered a 100% nucleotide identity. PRRSV-2 ORF5 gene sequence was classified as Lineage 5 RFLP 2-5-2, which had 99.0% similarity with the Ingelvac PRRS^®^ MLV vaccine virus. PRRSV Ingelvac MLV ORF5 CLAMP sequencing was conducted to rule out the presence of a wild-type PRRSV, with the detection of only the Ingelvac PRRSV MLV ORF5. Microscopic evaluation of the lung tissue found numerous bronchioles surrounded by moderate-sized cuffs of lymphocytes. Alveoli in affected areas commonly contained a moderate number of neutrophils. No remarkable lesions were identified in the spleen and lymph nodes. The observed changes are consistent with MHP infection with secondary bacterial bronchopneumonia consistent with the MHP detected in the lung *via* PCR. Although PRRSV-2 was detected in the lung *via* PCR at a low Ct (Ct 15.2), no lesions suggestive of viral interstitial pneumonia were apparent in the examined sections.

**Figure 1:**
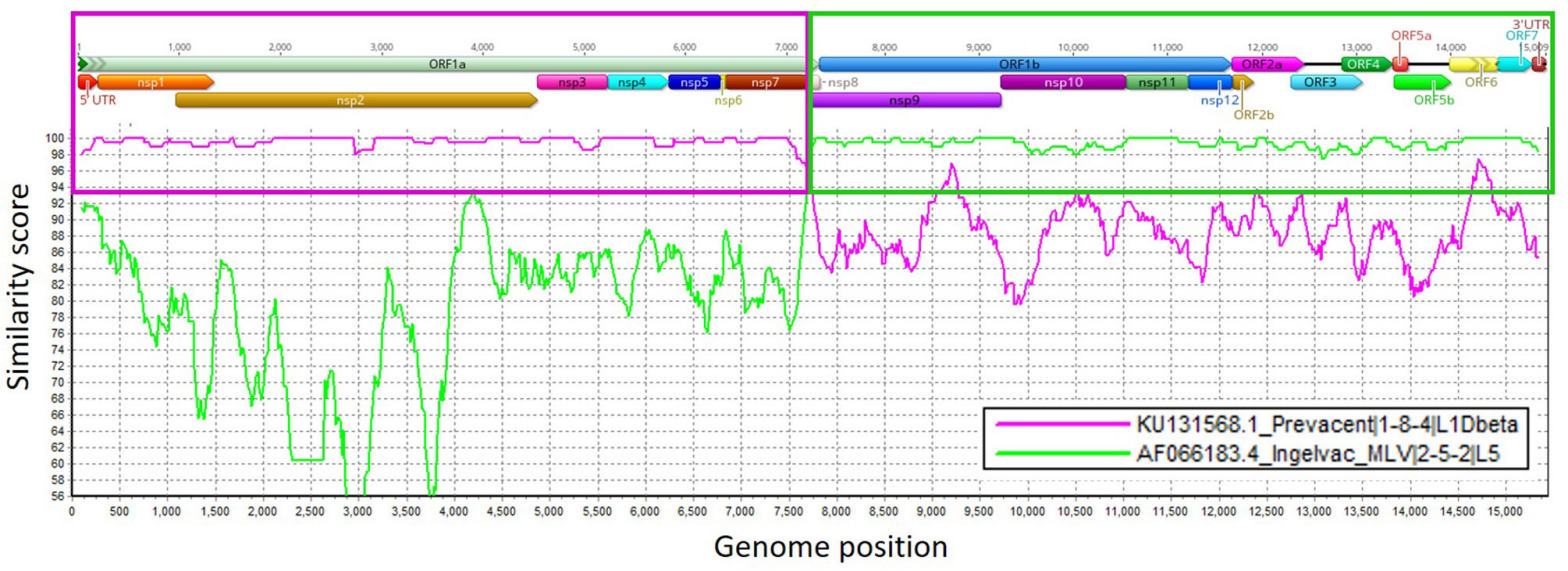
Similarity plot analysis using the isolate USA/IN105404/2021 strain as the query sequence against Ingelvac^®^ PRRS MLV and Prevacent^®^ PRRS. Genome regions (ORFs) and non-structural protein (NSP) genome regions of the recombinant strain are presented at the top of the plot. Purple and green boxes outline the recombination breakpoints, similarity scores, and genome regions that were derived from Prevacent^®^ PRRS (major) and Ingelvac^®^ PRRS MLV (minor) parental strains. The closer the similarity score is to 100 (y-axis), the more similar the compared strains are. Genome regions were plotted in Geneious, and recombination events were determined using RDP4 and confirmed in SimPlot (1).

**Figure 2:**
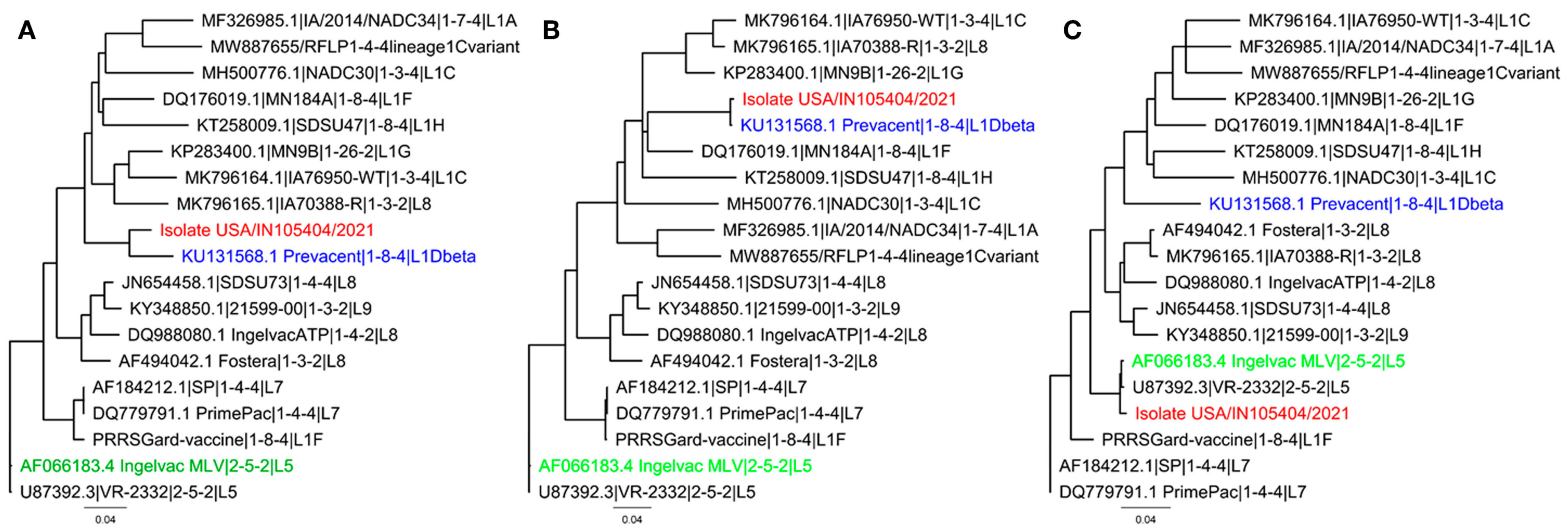
Neighbor-joining phylogenetic tree representing the recombinant isolate USA/IN105404/2021 (red), the major parental strain Prevacent^®^ PRRS (blue), and the minor parental strain Ingelvac^®^ PRRS MLV (green). Strains for additional PRRSV-modified live vaccines commercially available in the United States and other PRRSV-2 wild-type strains are presented as references. **(A)** Whole genome neighbor-joining phylogenetic tree; **(B)** neighbor-joining phylogenetic tree for the genome portion before the recombination breakpoint. **(C)** Neighbor-joining phylogenetic for the genome portion after the recombination breakpoint.

**Figure 3:**
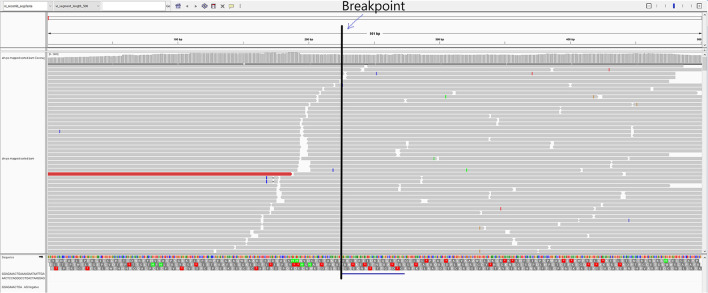
IGV representation of the mapped reads and virus isolate USA/IN105404/2021. The vertical line represents the recombination breakpoint identified at the 7,243 nt position. The reads marked before the breakpoint line were identical to the Prevacent^®^ PRRS vaccine strain, whereas the reads after that matched the Ingelvac^®^ PRRS MLV vaccine strain.

In the first passage (P0), a virus isolate was recovered from the lung sample in both MARC-145 and ZMAC cells. The isolate obtained in ZMAC cells (P0) was submitted for NGS examination, and a whole PRRSV genome was recovered from the isolate. Three genome fragments, i.e., contigs, were recovered from the indigenous lung sample. Contig length, nucleotide identity, and start and end point comparisons with the isolate USA/IN105404/2021 were as follows: (a) 3,467 base pairs (bp), 99.97%, position 61–3,527; (b) 3,152 bp, 99.94%, position 3,607–6,758; and (c) 8,204 bp, 100%, position 6,794–14,997. Recombination analysis indicated that the isolate was a recombinant between the Ingelvac PRRS^®^ MLV and Prevacent^®^ PRRS vaccine viruses. A putative recombination breakpoint was identified at position 7,664 of the isolate, which is located in the nsp8 encoding region (Figure 1). Compared with the PRRSV-2 VR-2332 referent strain, the breakpoint was located at position 7,634 without gaps in the VR-2332 strain (GenBank U87392.3). A neighbor-joining tree revealed that the recombinant virus had closer proximity with the Prevacent^®^ PRRS major parental strain at the whole genome level (Figure 2A) and the genomic region before the breakpoint (Figure 2B), whereas it had closer proximity with the Ingelvac^®^ PRRS MLV minor parental strain at the genomic region after the breakpoint (Figure 2C). Nucleotide dissimilarity for genes (ORFs) and non-structural proteins (NSP) with additional PRRS MLV vaccines and wild-type strains provided supporting evidence of recombination between Ingelvac^®^ PRRS MLV and Prevacent^®^ PRRS (Table 1). In addition, more than 1,000 raw sequencing reads from the whole genome sequencing of the isolate USA/IN105404/2021 crossing the breakpoint were identified and illustrated by using the Integrative Genomics Viewer (Figure 3). A high-level nucleotide identity for the ORF1b-ORF7 genes was also obtained between the isolate USA/IN105404/2021 and the wild-type PRRSV-2 VR-2332 strain. A high level of similarity with the VR-2332 strain was expected since the VR-2332 strain was the parental strain used to develop the Ingelvac^®^ PRRS MLV vaccine. The contribution of the wild-type VR-2332 strain in the recombination event is unlikely since this strain was recovered 30 years ago (3), and no history of contact or infection with wild-type PRRSV strain(s) has been previously identified in the pig herd examined in this study.

**Table 1:** Genome regions and nucleotide identity between isolate USA/IN105404/2021 and other vaccine and wild-type strains.

	**KU131568.1 Prevacent**	**AF066183.4 Ingelvac MLV**	**DQ779791.1 PrimePac**	**DQ988080.1 IngelvacATP**	**AF494042.1 Fostera**	**PRRSGard**	**U87392.3|VR-2332**	**AF184212.1| SP**	**DQ176019.1|MN184A**	**JN654458.1|SDSU73**	**KT258009.1|SDSU47**	**KP283400.1|MN9B**	**KY348850.1|21599-00**	**MH500776.1|NADC30**	**MK796164.1|IA76950-WT**	**MK796165.1|IA70388-R**	**MW887655/1-4-4L1Cvar.**	**MF326985.1|IA/2014/NADC34**
WGS	94.3	88.5	85.7	84.1	84.7	90.5	88.5	85.7	88.5	84.5	85.7	86.6	83.9	85.0	85.2	88.5	82.9	84.1
5‘UTR	97.9	91.1	90.0	88.5	89.5	90.0	91.1	90.0	91.1	87.9	89.5	90.4	87.9	91.6	88.7	89.2	91.3	91.6
ORF1a	99.5	83.1	84.8	82.1	82.2	85.1	83.2	84.8	87.7	82.3	83.7	85.3	82.1	82.9	83.8	83.4	82.6	84.5
nsp1	99.6	84.4	83.8	81.7	82.9	83.8	84.4	83.8	85.7	82.0	82.8	84.5	81.7	84.4	83.8	83.5	82.1	82.9
nsp2	99.6	85.8	85.1	79.2	79.5	86.2	80.4	85.1	87.5	79.4	81.1	83.9	79.0	80.7	82.0	81.8	82.7	83.6
nsp3	99.6	85.8	85.1	83.5	83.9	85.1	85.9	85.1	91.0	83.6	87.7	88.4	84.0	82.9	87.4	87.0	82.8	85.2
nsp4	99.7	85.1	84.3	86.0	84.6	84.5	85.1	84.3	92.2	86.4	89.4	89.7	86.1	83.2	86.9	87.1	82.8	88.7
nsp5	99.6	84.1	84.3	84.3	83.7	84.3	84.1	84.3	85.7	84.3	85.7	87.8	83.5	80.4	88.2	87.5	82.4	81.2
nsp6	100	93.8	91.7	91.7	93.8	91.7	93.8	91.7	93.8	91.7	93.8	97.9	91.7	91.7	93.8	93.8	89.6	89.6
nsp7	99.9	83.3	83.8	81.7	82.2	83.9	83.3	83.8	85.3	82.0	83.3	82.2	82.5	87.3	90.3	81.9	81.0	83.8
nsp8	96.3	91.9	89.6	91.1	90.4	89.6	91.6	89.6	89.6	88.9	86.7	87.4	88.9	90.4	88.1	89.6	87.4	89.6
ORF1b	89.1	99.5	95.7	92.5	92.6	95.8	99.3	95.7	88.5	92.8	87.7	87.0	92.4	87.1	86.2	92.6	86.6	86.8
nsp9	89.8	99.1	94.3	92.7	93.0	94.5	99.1	94.3	88.8	92.8	87.8	87.9	92.4	88.1	86.9	92.6	87.9	87.7
nsp10	88.3	98.9	96.6	92.4	91.8	96.5	98.7	96.6	88.0	92.9	86.8	86.6	92.4	85.3	85.7	92.2	85.8	86.1
nsp11	90.3	100	96.1	91.9	92.5	96.0	99.3	96.1	89.5	93.0	88.9	87.0	92.5	88.6	86.6	92.5	85.8	87.3
nsp12	88.4	99.4	97.0	91.9	93.0	97.0	99.1	97.0	87.0	91.3	87.4	84.1	90.8	87.3	84.1	92.8	84.4	87.3
ORF2a	90.9	99.2	94.4	94.2	95.1	94.2	99.1	94.4	90.4	93.9	88.0	90.0	92.7	87.3	87.8	94.6	87.3	89.3
ORF2b	89.2	98.7	95.5	92.8	93.7	95.5	98.7	95.5	91.9	92.3	88.3	91.4	89.6	90.5	88.7	93.2	90.5	91.3
ORF3	89.8	98.8	90.7	91.1	91.8	89.8	98.4	90.7	89.5	91.1	86.0	87.3	90.1	83.7	84.8	91.6	83.4	84.6
ORF4	88.5	99.3	93.1	90.9	92.2	89.6	99.3	93.3	89.2	92.2	89.4	87.5	91.4	86.6	86.8	92.6	85.5	87.2
ORF5b	86.2	99.0	91.2	89.9	91.8	85.9	99.3	91.2	85.9	90.3	84.7	86.7	88.1	85.4	85.2	91.6	86.4	88.1
ORF5a	87.8	98.7	93.0	90.4	92.3	85.9	99.4	93.0	86.5	91.7	88.5	88.5	84.6	87.2	86.5	92.3	89.7	89.1
ORF6	92.8	99.8	94.7	94.7	96.0	92.0	99.3	94.7	91.8	96.0	90.3	93.0	94.1	90.7	89.9	95.8	89.7	89.3
ORF7	91.4	99.7	96.0	92.9	94.6	93.3	99.7	96.0	93.3	94.9	82.7	90.9	94.1	93.0	91.0	94.1	89.8	91.1
3'UTR	87.9	98.6	96.5	91.5	93.6	92.8	98.6	96.5	91.5	95.0	93.0	91.5	93.5	90.8	88.7	93.6	91.5	92.2

Green-shaded cells identify the recombinant regions with the major parental strain (Prevacent^®^ PRRS) and minor parental strain (Ingelvac^®^ PRRS MLV).

## 4. Discussion

This study provides solid evidence for the emergence of a natural recombinant PRRSV-2 field strain derived from two commercially available PRRSV-2 MLV vaccines available in the USA. Even though PCR detected a high PRRSV viral load (Ct 15.2) in the lung suggesting the ability of this recombinant virus to replicate in the host, microscopic lesions associated with PRRSV interstitial pneumonia were not observed. Under the conditions of this study, it appears that the recombinant strain was not virulent, although it is also possible that previous vaccination may provide protective immunity. Nonetheless, the pathogenicity of this recombinant virus needs to be unequivocally determined by an experimental inoculation study in pigs.

Porcine reproductive and respiratory syndrome virus (PRRSV) has previously been shown to persist for long periods in infected pigs (45, 46). Under experimental conditions, PRRSV was recovered from tonsils at 150 days post-infection and had the ability to develop viremia and seroconversion in a subsequent bioassay study (47). Co-infection with two (or more) strains in the same host and the same cell at the same time is required for a recombination event to occur (26). Nevertheless, exposure to both vaccine viruses has occurred in this pig flow, i.e., Ingelvac^®^ PRRS MLV in the breeding herd and Prevacent^®^ PRRS in the piglets. Recovery of the recombinant isolate occurred 11 months after the last Ingelvac^®^ PRRS MLV exposure in the breeding herd and approximately 4 months after exposure of the piglets to Prevacent^®^ PRRS. In another study in a breeding herd subsequently exposed to Ingelvac^®^ PRRS MLV, detection of PRRSV in processing fluid samples was intermittent and associated with stress factors, e.g., the introduction of additional pathogens, after the third whole-herd exposure to the vaccine virus that occurred at 39 weeks after initial exposure (22). It would be possible that the recombinant PRRSV identified in this study occurred after some Ingelvac^®^ PRRS MLV leakage from the breeding herd, providing the opportunity for co-circulation of both strains in the wean-to-finish population and creating the conditions necessary for a recombination event. The possibility that this recombination event of two PRRSV MLV strains could have occurred in another site or a different population of pigs and been subsequently introduced into the herd has not been explored.

Considering that co-infection with two (or more) strains in the same host and the same cell simultaneously is required for recombination events, consistent usage of a single PRRSV MLV vaccine may help prevent MLV vaccine-associated recombination events. In the case of this study, if the PRRSV MLV exposure strategy used in pigs at the wean-to-finish site was with Ingelvac^®^ PRRS MLV or if the breeding herd was exposed to Prevacent^®^ PRRS, keeping the vaccination strategies consistent in both populations, this recombination event could have been potentially prevented. Live virus inoculation (LVI) is still a practice used in the USA for breeding herd exposure, and further testing of LVI material prior to its use, e.g., using an NGS approach to investigate the presence of a single or multiple wild-type PRRSV strain(s) is recommended. Not using LVI material containing multiple PRRSV strains can reduce the intentional exposure of pigs to more than one PRRSV strain, decreasing the opportunity for co-infection of the same host and cell with distinct PRRSV wild-type or MLV vaccine strains. Routine testing of gilts to avoid the introduction of PRRSV-infected gilts with a farm-distinct wild-type strain(s) should be encouraged. Moreover, whenever possible, reducing the commingling of piglets infected with distinct PRRSV strains can help reduce the emergence of new recombinant strains. These are examples of measures that could help reduce the opportunity presented by emerging virulent PRRSV recombinant strains such as the L1C variant RFLP 1-4-4 strain that emerged in the USA in recent years (48, 49).

In this study, PCR results were initially confusing due to the discrepancy between screening PCR, vaccine-like PCR results, and the ORF5 sequence obtained from the lung. This prompted virus isolation and further investigation using NGS on the virus isolate and the original lung sample. The whole genome sequence was obtained from the isolate USA/IN105404/2021 and the three contig sequences were obtained from the indigenous lung sample *via* NGS to help align with the PRRSV screening PCR, vaccine-specific PCR, and ORF5 Sanger sequencing results. The Ingelvac MLV-like PCR yielded a negative result, whereas the Elanco Prevacent-like PCR was positive. Both of these vaccine-like PCRs target the nsp2 region of the genome, and that portion of the isolate USA/IN105404/2021 genome had high nucleotide identity (99.6%) with the Prevacent^®^ PRRS vaccine sequence. In contrast, the PRRSV Ingelvac MLV ORF5 CLAMP and Sanger sequencing targeting the ORF5 gene recovered an Ingelvac MLV strain consistent with the whole genome sequence, considering that a portion of the whole genome (ORF1b–ORF7) had a high nucleotide identity (99%) with the Ingelvac^®^ PRRS MLV sequence. The conflicting results between a Prevacent^®^ PRRS PCR positive outcome and detection of an Ingelvac MLV ORF5 sequence, together with the additional NGS results, clearly suggested the presence of a recombinant PRRSV strain in the lung sample and the derived isolate.

The process of first performing virus isolation and then applying NGS to the isolate is recommended to obtain more specific sequencing reads when performing NGS, making the results more conclusive for the presence of a single virus representing the recombinant strain. An indigenous clinical sample could potentially contain more than one PRRSV strain and could produce genetic sequences of either strain when submitted for Sanger or NGS sequencing. A virus isolation process favors the selection of one strain (50). Therefore, the NGS performed on a virus isolate made it conclusive for recovering and discerning the presence of a single recombinant strain in the sample. Although plaque-purification of a virus isolate was not attempted in this study, raw sequencing reads crossing the breakpoint were identified with one terminus most closely related to the Ingelvac PRRS^®^ MLV sequence and the other terminus most closely related to the Prevacent^®^ PRRS sequence, providing direct evidence that the assembled recombinant sequence was from a single molecule but not from two different genomes.

These findings contribute to our understanding of PRRSV evolution and epidemiology. Compared to the ORF5 sequence, which is only 603 bp and comprises ~ 4% of the entire genome, using the full-length PRRSV genome for epidemiological investigations has a competitive advantage to better characterize PRRSV strains across all genome regions. Full-length genome sequencing is necessary to detect recombination events. The NGS technology makes it more feasible nowadays to determine the full-length PRRSV genome sequences at a reasonable cost. The use of NGS also improves epidemiological investigations when whole genomes are compared versus the exclusive use of the ORF5 gene sequence (23).

## Data availability statement

The PRRSV whole-genome sequence isolate USA/IN105404/2021 has been deposited in GenBank under accession no. OQ145436 (https://www.ncbi.nlm.nih.gov/nuccore/OQ145436). The Illumina MiSeq reads have been deposited in the SRA under BioProject accession no. SRR23173625 (https://www.ncbi.nlm.nih.gov/sra/?term=SRR23173625).

## Ethics statement

This research was conducted on clinical diagnostic samples that had been submitted for diagnostic testing. Written informed consent was obtained from the owners for the participation of their animals in this study.

## Author contributions

GT, DM, AW, JS, and PG contributed to the conception and design of the study. GT, MZ, and AS organized the database and performed the statistical analysis. GT wrote the first draft of the manuscript. KK, JZ, DM, and GL wrote sections of the manuscript. All authors contributed to the manuscript revision, read, and approved the submitted version.
